# Cuproptosis causes meiotic metaphase I arrest by disrupting mitochondrial functions in oocytes

**DOI:** 10.1038/s41420-026-03168-x

**Published:** 2026-05-23

**Authors:** You-Hui Lu, Can Wang, Lei-Ning Chen, Li-Tao Yi, Ke Xu, Xu-Feng Li, Shu-Chen Liu, Xiao-Yi Chen, Yi-Xiao Li, Qing-Yuan Sun, Qiong Wang, Tie-Gang Meng

**Affiliations:** 1 Department of Obstetrics and Gynecology, Reproductive Medical Center, First Affiliated Hospital of Sun Yat-sen University, Guangzhou, China; 2 Guangzhou Key Laboratory of Metabolic Diseases and Reproductive Health, Guangdong-Hong Kong Metabolism & Reproduction Joint Laboratory, Reproductive Medicine Center, the Affiliated Guangdong Second Provincial General Hospital of Jinan University, Guangzhou, China; 3 State Key Laboratory of Female Fertility Promotion, Center for Reproductive Medicine, Department of Obstetrics and Gynecology, Peking University Third Hospital, Beijing, China; 4 The Second School of Clinical Medicine, Southern Medical University, Guangzhou, China; 5 School of Basic Medical Sciences, Southern Medical University, Guangzhou, China; 6 Key Laboratory of Regenerative Medicine of Ministry of Education, College of Life Science and Technology, Jinan University, Guangzhou, China; 7 Guangdong Provincial Key Laboratory of Reproductive Medicine, First Affiliated Hospital of Sun Yat-sen University, Guangzhou, China

**Keywords:** Cell death, Germline development

## Abstract

Proper oocyte maturation is critical for female fertility, yet whether cuproptosis, a recently identified copper-dependent cell death pathway, affects meiotic maturation remains unknown. Here, we show that Cu(II)-elesclomol (ELC-Cu(II)) treatment induces dose-dependent metaphase I arrest of mouse oocytes. This arrest results from spindle assembly checkpoint activation caused by defective spindle organization and impaired kinetochore-microtubule attachments. We demonstrate that ELC-Cu(II) triggers changes in canonical cuproptosis markers, including intracellular copper accumulation, FDX1 downregulation, and protein aggregation. Meanwhile, treated oocytes exhibit mitochondrial dysfunction characterized by reduced membrane potential and decreased ATP levels. Integrated transcriptomic and proteomic profiling reveals a predominantly post-transcriptional response, with 223 differentially expressed proteins, while transcriptomic profiles show minimal changes. Pathway analysis identifies dysregulation of lipoic acid metabolism and iron-sulfur cluster biosynthesis as key features. Targeted knockdown of the key lipoyltransferase LIPT1 fails to rescue the meiotic defect, whereas supplementation with the NAD^+^ precursor nicotinamide mononucleotide (NMN) improves mitochondrial function and partially restores polar body extrusion. These findings establish cuproptosis as a mechanism linking copper toxicity to mitochondrial impairment and meiotic failure in oocytes, and suggest NAD^+^ metabolism as a potential therapeutic target for protecting oocyte quality.

**Figure:**
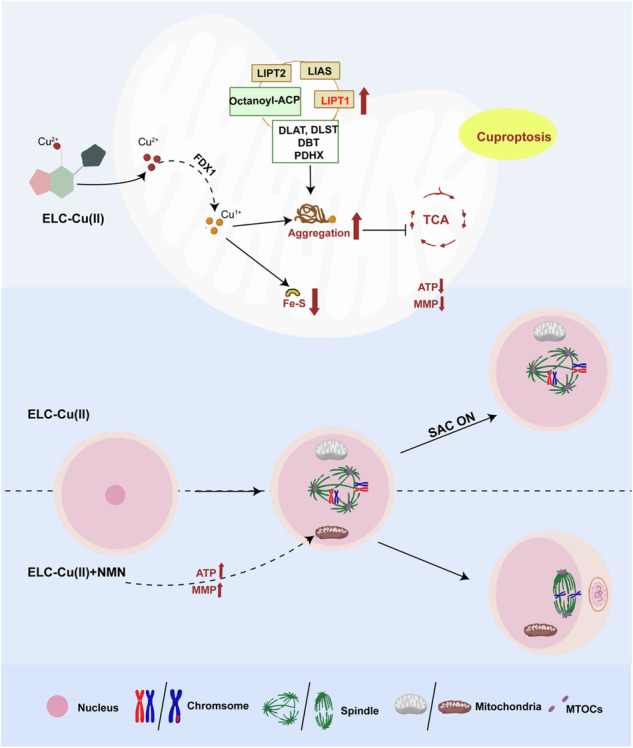
Cuproptosis, via copper accumulation and FDX1 loss, disrupts critical metabolic pathways (such as lipoic acid metabolism and iron-sulfur cluster biosynthesis), causing mitochondrial dysfunction and oocyte meiotic arrest. NMN supplementation effectively mitigates this arrest by restoring cellular energy metabolism and rescuing maturation.

Cuproptosis, via copper accumulation and FDX1 loss, disrupts critical metabolic pathways (such as lipoic acid metabolism and iron-sulfur cluster biosynthesis), causing mitochondrial dysfunction and oocyte meiotic arrest. NMN supplementation effectively mitigates this arrest by restoring cellular energy metabolism and rescuing maturation.

## Introduction

Oocyte maturation determines the success of fertilization, early embryonic development and the outcomes of assisted reproductive technologies (ART) [1]. The first meiotic division is particularly susceptible to error, with chromosome segregation defects frequently leading to aneuploidy, which accounts for a substantial proportion of infertility, pregnancy loss or congenital disorders [2]. Unlike mitotic cells, oocytes rely on a finite pool of maternally inherited mRNAs and proteins, making them highly dependent on post-transcriptional regulation [3] and vulnerable to stressors that disrupt proteostasis or organellar function.

Mitochondria play an essential role in oocyte maturation, providing the ATP required for spindle assembly, chromosome segregation, and cytokinesis [4, 5]. Mitochondrial dysfunction has been implicated in oocyte aging and various forms of regulated cell death, including apoptosis [6] and ferroptosis [7]. However, the full spectrum of stress pathways that compromise oocyte quality remains incompletely defined. Recently, a novel form of copper-dependent cell death termed cuproptosis was identified in cancer cells [8, 9]. Unlike other cell death modalities, cuproptosis is triggered by excess intracellular copper, which binds to lipoylated enzymes of the tricarboxylic acid (TCA) cycle, causing their aggregation and proteotoxic stress. This process leads to iron-sulfur (Fe-S) cluster depletion and mitochondrial dysfunction. The pathway is regulated upstream by the enzyme ferredoxin 1 (FDX1), which directly reduces copper ions (from Cu²⁺ to Cu⁺) and mediates protein lipoylation [10, 11], thereby sensitizing cells to copper-induced toxicity.

Given the oocyte’s heavy reliance on mitochondrial metabolism [12] and proteostasis [13], we hypothesized that the oocyte may be particularly vulnerable to cuproptosis, which may represent a previously unrecognized mechanism of meiotic failure. Despite the surge of cuproptosis studies in oncology, its function in relevance to physiological systems—particularly in post-mitotic cells such as the oocyte—remains largely unexplored.

Here, we investigate the role of cuproptosis in mouse oocyte meiotic maturation using the cuproptosis inducer Cu(II)-elesclomol(ELC-Cu(II)). Through a combination of live-cell imaging, immunofluorescence, and multi-omics analysis, we characterize the cellular and molecular cascade following cuproptosis induction and identify potential therapeutic targets to mitigate copper-induced oocyte damage.

## Results

### ELC-Cu(II) treatment impairs meiotic maturation of mouse oocytes

**Fig. 1:**
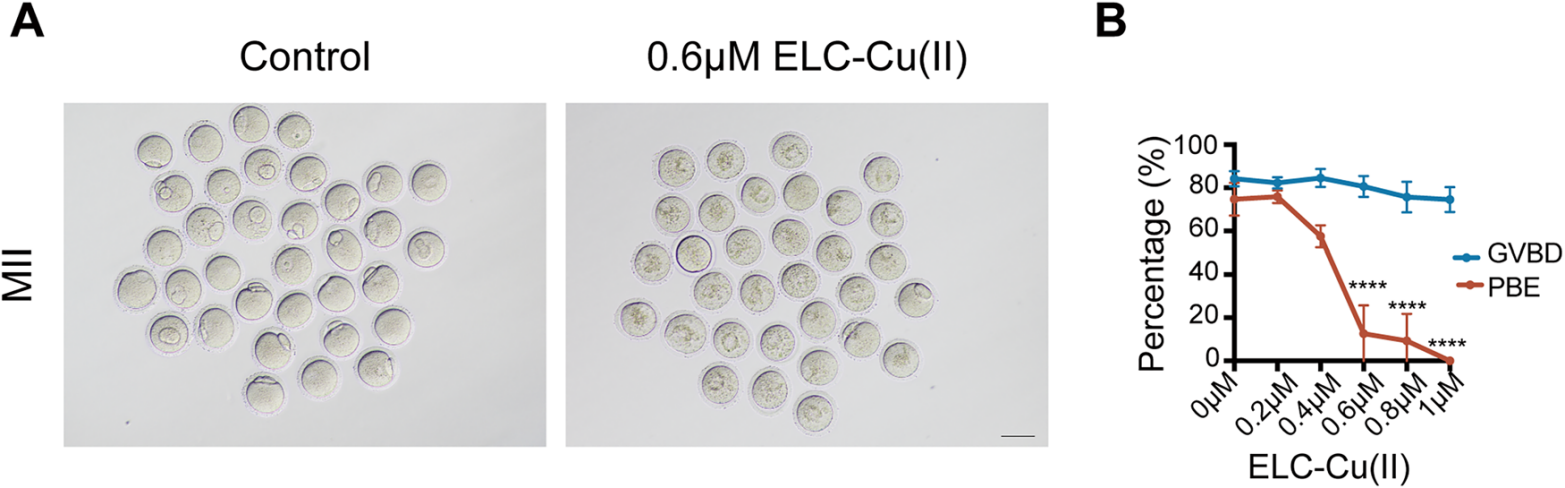
The effect of ELC-Cu(II) treatment on the meiotic progression of mouse oocytes. **A** Representative images of oocytes at the metaphase II (MII) stage at 14 h post-release from 3-isobutyl-1-methylxanthine (IBMX) in the control and 0.6 μM ELC-Cu(II) groups. Scale bar =100 um. **B** The rate of first polar body extrusion (PBE) was quantified for oocytes in the control group (*n* = 99) and in groups treated with 0.2 μM (*n* = 65), 0.4 μM (*n* = 121), 0.6 μM (*n* = 130), 0.8 μM (*n* = 133), and 1 μM (*n* = 100) ELC-Cu(II). Data are presented as the mean percentage ± SEM from at least three independent experiments. ****P < 0.0001.

To investigate whether cuproptosis affects oocyte meiotic maturation, we treated fully-grown oocytes (FGO) with increasing concentrations (0, 0.2, 0.4, 0.6, 0.8, and 1 μM) of the cuproptosis inducer Cu(II)-Elesclomol(ELC-Cu(II)) and monitored meiotic progression. Germinal vesicle breakdown (GVBD), which marks the G2/M transition and meiotic resumption, occurred at comparable rates across all treatment groups (Fig. 1B), indicating that ELC-Cu(II) does not prevent meiotic resumption. However, first polar body extrusion (PBE), which reflects successful completion of meiosis I, was significantly reduced in a dose-dependent manner. The PBE rate decreased from 74.7 ± 3.8% in control oocytes (*n* = 99) to 11.3 ± 5.2% in oocytes treated with 0.6 μM ELC-Cu(II) (n = 130; *P* < 0.0001; Fig. 1A, B). Higher concentrations (0.8 and 1.0 μM) produced comparable inhibition, suggesting that 0.6 μM approaches the maximal effect. Based on these results, we selected 0.6 μM ELC-Cu(II) for all subsequent mechanistic studies.

### ELC-Cu(II) treatment induces metaphase I arrest through spindle assembly checkpoint activation

**Fig. 2:**
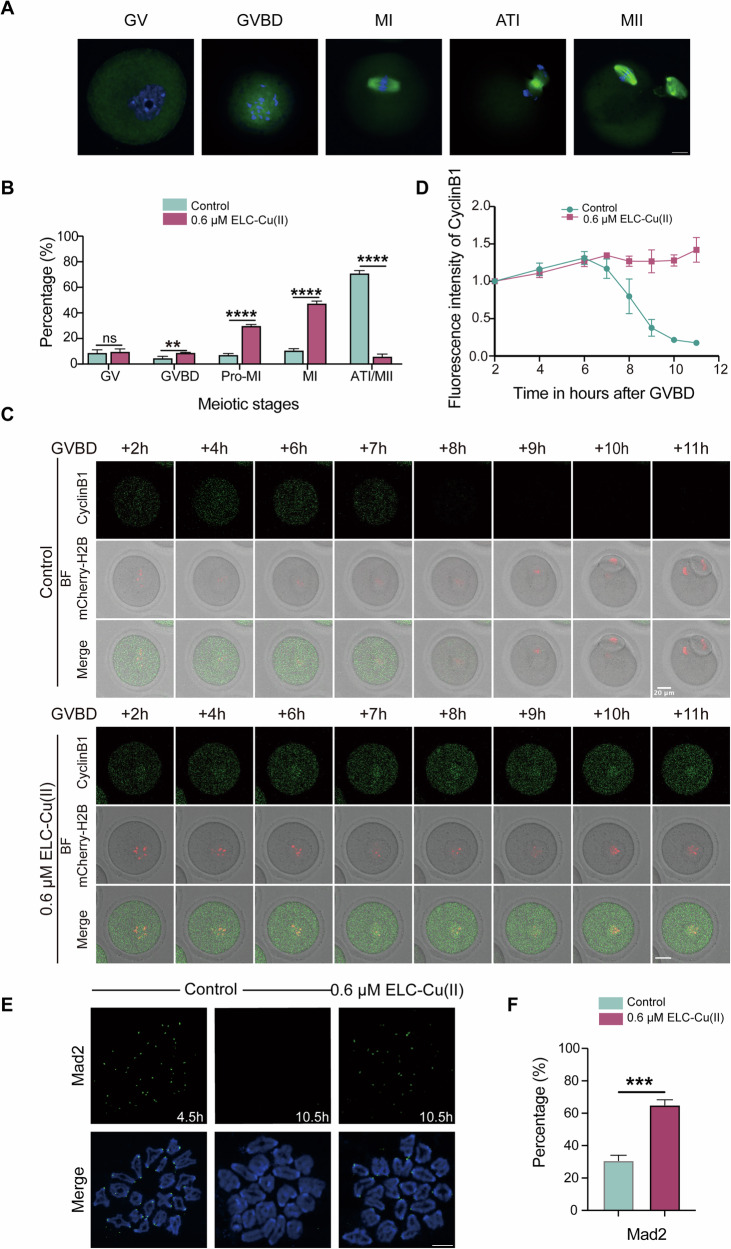
ELC-Cu(II) treatment disrupts cell cycle progression during oocyte maturation. **A** Representative immunofluorescence images of spindle and chromosome morphology at the GV, GVBD, MI, ATI, and MII.α-tubulin are shown in green, DNA are shown in blue. Scale bar = 20 μm. **B** Quantification of the percentage of oocytes arrested at different developmental stages in the control (*n* = 99) and 0.6 μM ELC-Cu(II)-treated (*n* = 130) groups. **C** Representative time-lapse images showing the degradation of exogenous cyclin B1-GFP in control and 0.6 μM ELC-Cu(II)-treated oocytes after GVBD. Images are overlaid with mCherry, GFP and BF channels. Scale bars: 20 μm. **D** Fluorescence intensity of cyclin B1-GFP in control(*n* = 34) and 0.6 μM ELC-Cu(II)-treated oocytes(*n* = 28) every 60 min. **E** Representative fluorescence images showing the localization of Mad2 on chromosomes in control and 0.6 μM ELC-Cu(II)-treated oocytes at the prometaphase I (4.5 h) and ATI stages (10.5 h). Mad2 is shown in green, and DNA is shown in blue. Scale bar = 20 μm. **F** The proportion of oocytes with Mad2 present on chromosomes at the ATI stage was quantified for the control (n = 96) and 0.6 μM ELC-Cu(II) (*n* = 106) groups. Data in (**B**),(**D**), and (**F**) are presented as the mean ± SEM from at least three independent experiments. **P < 0. 01; ***P < 0.001; ****P < 0.0001; ns, not significant.

To characterize the stage at which meiosis is disrupted, we examined oocyte cell cycle progression 14 h after release from IBMX by classifying oocytes according to their chromosome status and spindle morphology. While the majority of control oocytes (78.39 ± 2.73%) progressed to anaphase-telophase I/metaphase II (ATI/MII) stages, ELC-Cu(II)-treated oocytes predominantly arrested at earlier stages (Fig. 2A, B). Specifically, we observed significantly higher proportions of oocytes at GVBD (8.91 ± 0.25% vs 4.70 ± 1.35%, *P* < 0.01), prometaphase I (29.87 ± 0.97% vs 7.22 ± 1.00%, *P* < 0.0001), and metaphase I (47.37 ± 1.76% vs 10.69 ± 1.38%, *P* < 0.0001) in the ELC-Cu(II) group compared to controls.

To investigate the mechanisms underlying the observed arrest at prometaphase I or metaphase I, we monitored the dynamic behavior of Cyclin B1 (CCNB1) as a readout of APC/C activity. Given that CCNB1 degradation is a prerequisite for the metaphase-to-anaphase transition, we tracked its expression level and degradation kinetics following GVBD by live-cell imaging. Whereas control oocytes exhibited the expected gradual decline in CCNB1 fluorescence intensity, CCNB1 levels remained persistently elevated and failed to undergo timely degradation in ELC-Cu(II)-treated oocytes (Fig. 2C, D), indicative of impaired APC/C-mediated proteolysis. We therefore asked whether this aberrant CCNB1 stabilization was attributable to persistent activation of the spindle assembly checkpoint (SAC). Mad2, a core SAC component, is recruited to unattached kinetochores where it undergoes conformational changes that enable it to sequester Cdc20, thereby inhibiting the anaphase-promoting complex/cyclosome (APC/C) and blocking progression to anaphase [14]. To test whether SAC activation underlies the observed arrest, we examined Mad2 localization by immunofluorescence. In control oocytes at the anaphase-telophase I (ATI) stage, Mad2 signals were minimal, consistent with satisfied checkpoint and anaphase progression (Fig. 2E, F). In contrast, ELC-Cu(II)-treated oocytes exhibited persistent Mad2 signals on chromosomes, indicating sustained SAC activation (Fig. 2E, F). Quantification revealed that 63.21 ± 2.89% of ELC-Cu(II)-treated oocytes retained kinetochore-associated Mad2, compared to 31.25 ± 3.42% in control oocytes at comparable stages (*P* < 0.0001; Fig. 2F). These findings demonstrate that ELC-Cu(II) treatment activates the SAC, thereby preventing progression beyond metaphase I in mouse oocytes.

### ELC-Cu(II) treatment disrupts spindle organization and chromosome alignment

**Fig. 3:**
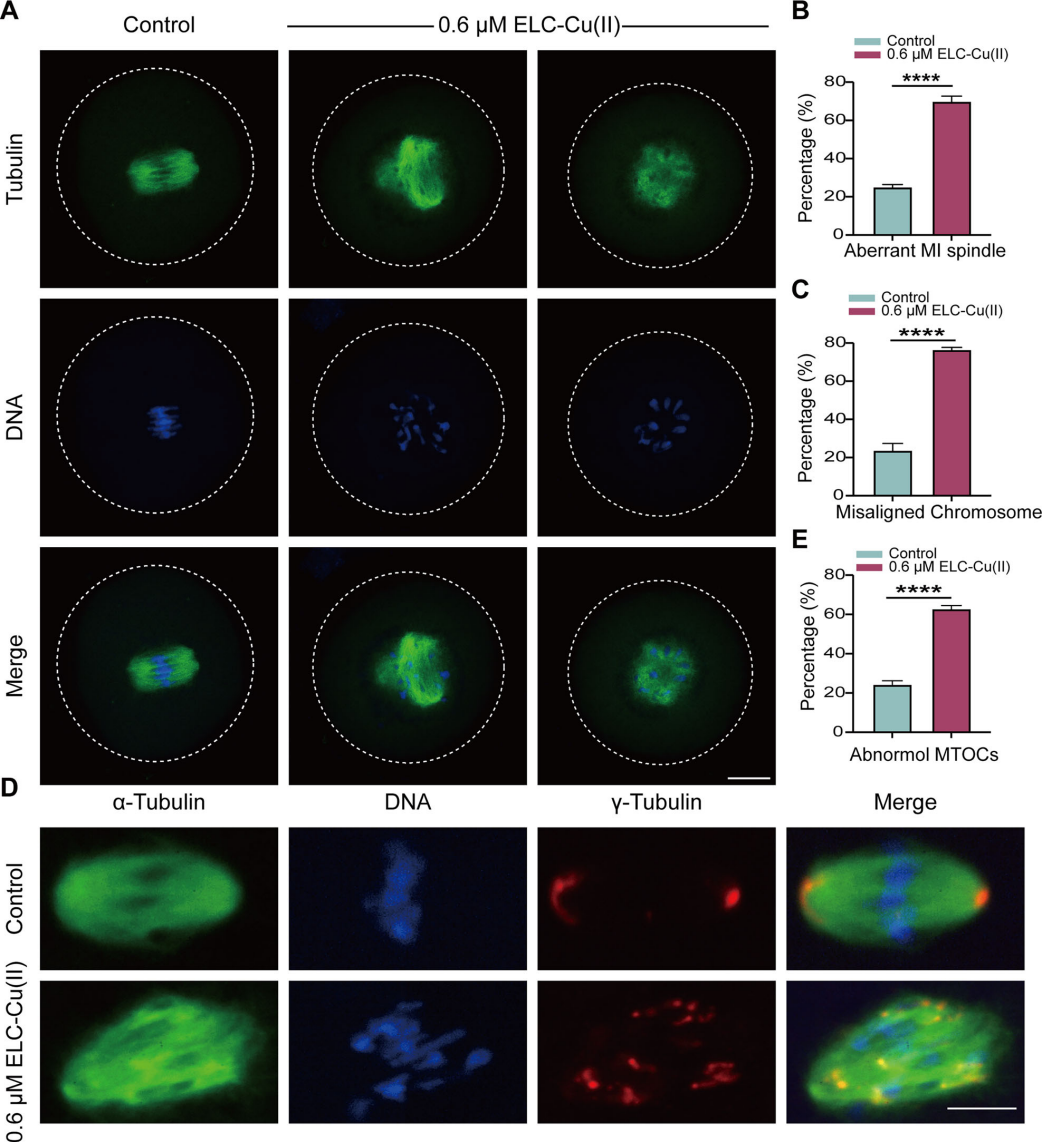
ELC-Cu(II) treatment impairs spindle organization and chromosome alignment in mouse oocytes. **A** Representative immunofluorescence images of spindle morphology and chromosome alignment in control and 0.6 μM ELC-Cu(II)-treated oocytes at the MI stage. Scale bar =10 μm. **B** Quantification of the percentage of oocytes with abnormal spindles in the control (*n* = 84) and 0.6 μM ELC-Cu(II)-treated groups (*n* = 89). **C** Quantification of the percentage of oocytes with misaligned chromosomes in the control (n = 84) and 0.6 μM ELC-Cu(II)-treated (*n* = 89) groups. **D** Representative immunofluorescence images of γ-tubulin localization, in control and 0.6 μM ELC-Cu(II)-treated oocytes at the MI stage. Scale bar = 10 μm. **E** Quantification of the percentage of oocytes with abnormal MTOCs in the control (*n* = 94) and 0.6 μM ELC-Cu(II)-treated (*n* = 88) groups. Data in (**B**), (**C**), and (**E**) are presented as the mean percentage± SEM from at least three independent experiments. ****P < 0.0001.

Given that defective spindle assembly is a primary trigger of SAC activation, we subsequently examined spindle morphology in ELC-Cu(II)-treated oocytes at the MI stage. Oocytes were immunostained with an anti-α-tubulin antibody to visualize microtubule organization and co-stained with DAPI to assess chromosome alignment. As shown in Fig. 3A, B, control oocytes displayed well-organized barrel-shaped spindles with chromosomes properly aligned at the metaphase plate (Fig. 3A). In contrast, ELC-Cu(II)-treated oocytes exhibited a significantly higher incidence of spindle abnormalities, including disorganized microtubule arrays, multipolar spindles, and abnormal spindle morphology (Fig. 3A, B). Correspondingly, chromosome misalignment was observed in ELC-Cu(II)-treated oocytes compared to controls (Fig. 3C). Since microtubule-organizing centers (MTOCs) are essential for proper spindle assembly in oocytes, we examined the distribution of γ-tubulin, a core MTOC component. In control oocytes, γ-tubulin localized to distinct foci at the spindle poles, consistent with proper MTOC organization (Fig. 3D). However, in ELC-Cu(II)-treated oocytes, γ-tubulin signals were diffuse and failed to concentrate at spindle poles, with 61.36 ± 3.18% of oocytes displaying abnormal MTOC organization compared to 21.28 ± 2.84% in controls (*P* < 0.0001; Fig. 3E). Taken together, these data suggest that ELC-Cu(II) treatment impairs MTOC function, leading to defective spindle assembly and chromosome misalignment during oocyte meiosis.

### ELC-Cu(II) treatment destabilizes kinetochore-microtubule attachments

**Fig. 4:**
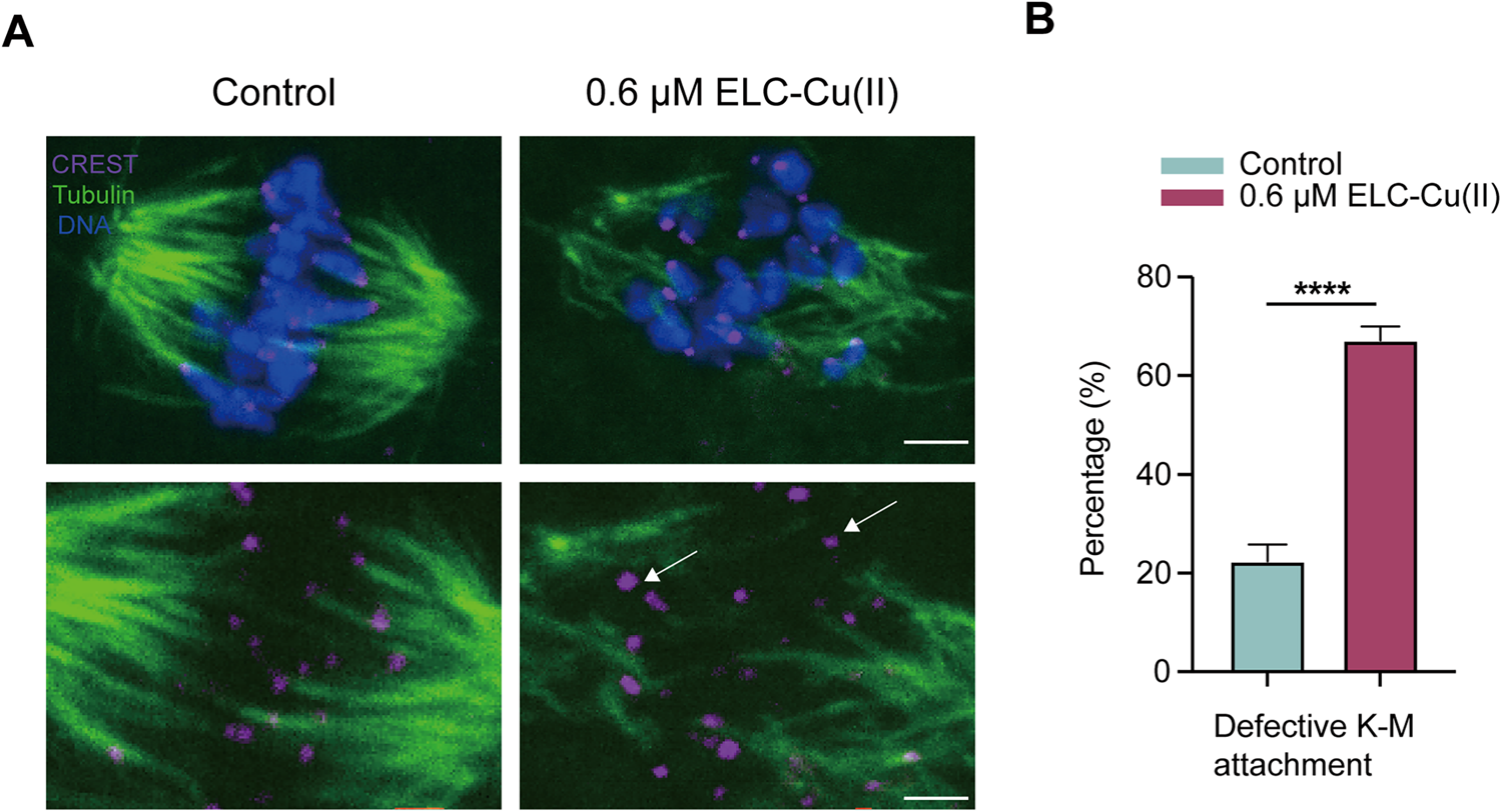
ELC-Cu(II) treatment disrupts kinetochore-microtubule attachments in mouse oocytes. **A** Representative immunofluorescence images of kinetochores and microtubule fibers in control and 0.6 μM ELC-Cu(II)-treated oocytes at the MI stage. To visualize stable K-MT attachments, non-kinetochore microtubules were depolymerized by incubating oocytes in pre-cooled M2 medium on ice for 5 min prior to fixation. White arrows indicate kinetochores that are not properly attached to spindle microtubules. Scale bars = 5 μm and 3.5 μm. **B** Quantification of the percentage of oocytes with unattached kinetochores in the control (*n* = 77) and 0.6 μM ELC-Cu(II)-treated (*n* = 79) groups. Data are presented as the mean ± SEM from at least three independent experiments. ****P < 0.0001.

Aberrant spindle organization and chromosome misalignment are typically linked to defective kinetochore-microtubule (K-MT) attachments. To assess attachment stability, oocytes were subjected to a cold-treatment assay in which they were exposed to 4 °C to depolymerize unstable microtubules, leaving only stable K-MT attachments intact. Oocytes were then immunostained with an α-tubulin antibody to visualize microtubules and with ACA to mark kinetochores. In control oocytes, the majority of microtubule fibers remained attached to the kinetochores following cold treatment, indicating stable K-MT connections (Fig. 4A, B). In contrast, ELC-Cu(II)-treated oocytes exhibited significantly more unattached kinetochores, indicating that the K-MT attachments were less stable and more susceptible to cold-induced depolymerization (Fig. 4A, B). These unattached kinetochores appeared as ACA-positive signals lacking associated microtubule fibers (indicated by arrows in Fig. 4A). These findings demonstrate that ELC-Cu(II) treatment destabilizes kinetochore-microtubule attachments. This instability, which is consistent with the observed spindle defects and chromosome misalignment, provides a mechanistic explanation for the sustained activation of the SAC.

### ELC-Cu(II) treatment induces changes in canonical cuproptosis markers in mouse oocytes

**Fig. 5:**
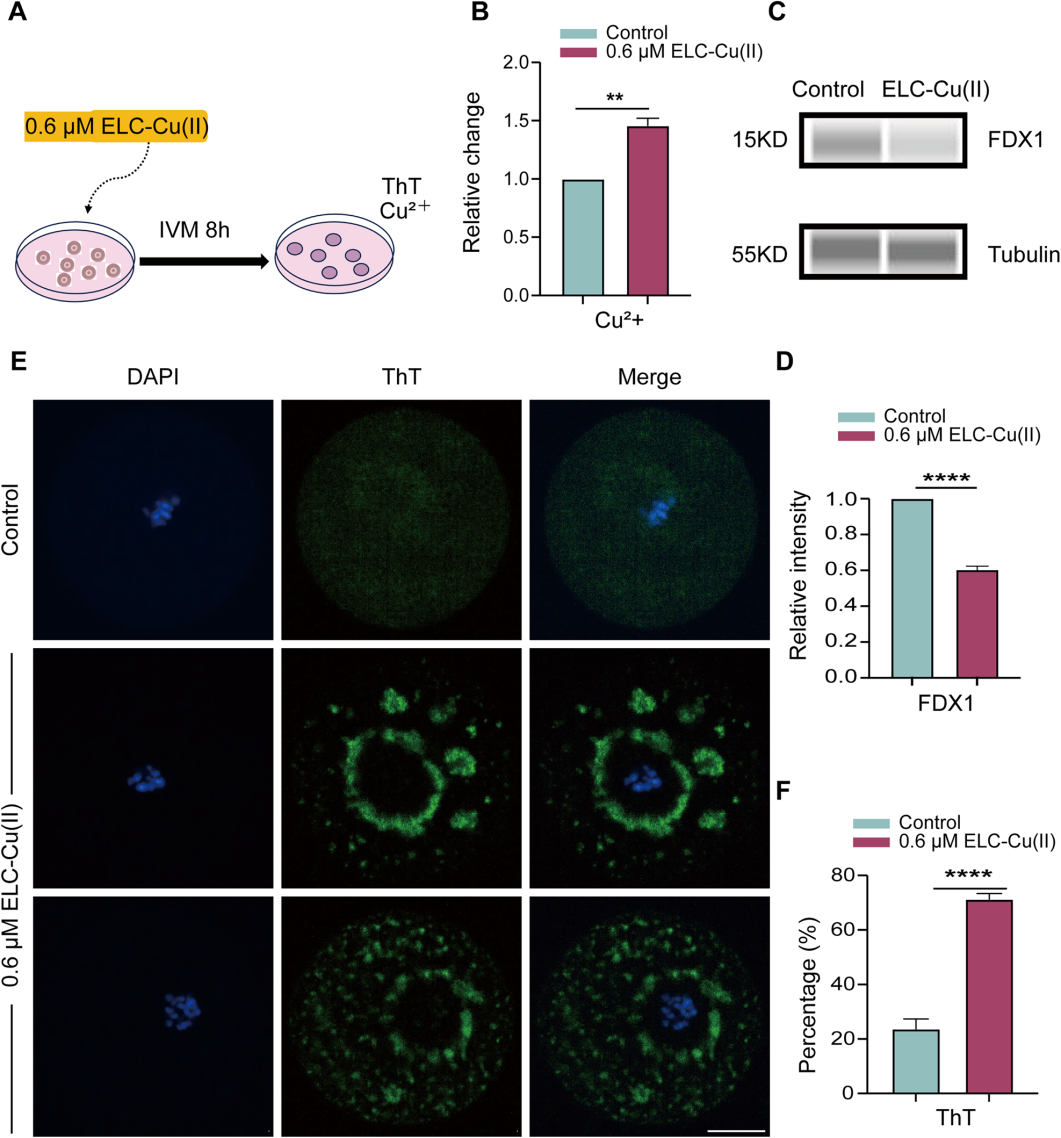
ELC-Cu(II) treatment induces cuproptosis signaling in mouse oocytes. **A** Schematic for detecting Cu²⁺ and ThT aggregates. **B** Quantification of relative intracellular Cu^2+^ levels in control (*n* = 60) and 0.6 μM ELC-Cu(II)-treated (*n* = 60) oocytes at MI stage. **C** Immunoblot analysis of FDX1 protein levels in control and 0.6 μM ELC-Cu(II)-treated oocytes. β-tubulin served as the loading control. **D** Quantification of FDX1 protein levels normalized to β-tubulin. **E** Representative fluorescence images of ThT staining in control and 0.6 μM ELC-Cu(II)-treated oocytesat the MI stage. Scale bar = 20 μm. **F** Quantification of the percentage of oocytes exhibiting abnormal ThT aggregates in the control (*n* = 48) and 0.6 μM ELC-Cu(II)-treated (*n* = 45) groups. Data in (**B**), (**D**) and (**F**) are presented as the mean ± SEM from at least three independent biological replicates. **P < 0.01; ****P < 0.0001.

To confirm that the observed meiotic defects result from cuproptosis rather than nonspecific copper toxicity, we examined the defining molecular hallmarks of this cell death pathway. Cuproptosis is characterized by three key features: intracellular copper accumulation, downregulation of ferredoxin 1 (FDX1), and proteotoxic stress resulting from aggregation of lipoylated proteins [15]. First, we measured intracellular copper levels using a copper ion concentration kit. ELC-Cu(II) treatment significantly elevated copper content in MI-stage oocytes to 1.459 ± 0.043-fold relative to controls (*P* < 0.01; Fig. 5A, B), confirming effective copper delivery into cells. Second, we assessed FDX1 protein expression, as FDX1 is an upstream regulator of cuproptosis whose downregulation is a characteristic feature of this pathway. Using automated capillary electrophoresis (Jess system), we detected a significant reduction in FDX1 protein levels in ELC-Cu(II)-treated oocytes compared to controls (relative expression: 0.604 ± 0.014, *P* < 0.01; Fig. 5C, D). Third, we examined proteotoxic stress by detecting protein aggregates with Thioflavin T (ThT), a fluorescent dye that binds to β-sheet-rich protein aggregates. While only 23.76 ± 2.10% of control oocytes (n = 48) displayed ThT-positive signals, 71.33 ± 1.18% of ELC-Cu(II)-treated oocytes (n = 45) exhibited prominent ThT-positive aggregates (*P* < 0.0001; Fig. 5E, F), indicating widespread protein aggregation. Together, these results demonstrate that ELC-Cu(II) treatment induces the canonical molecular signatures of cuproptosis—copper accumulation, FDX1 downregulation, and proteotoxic stress—in mouse oocytes.

### ELC-Cu(II) treatment disrupts mitochondrial distribution and function

**Fig. 6:**
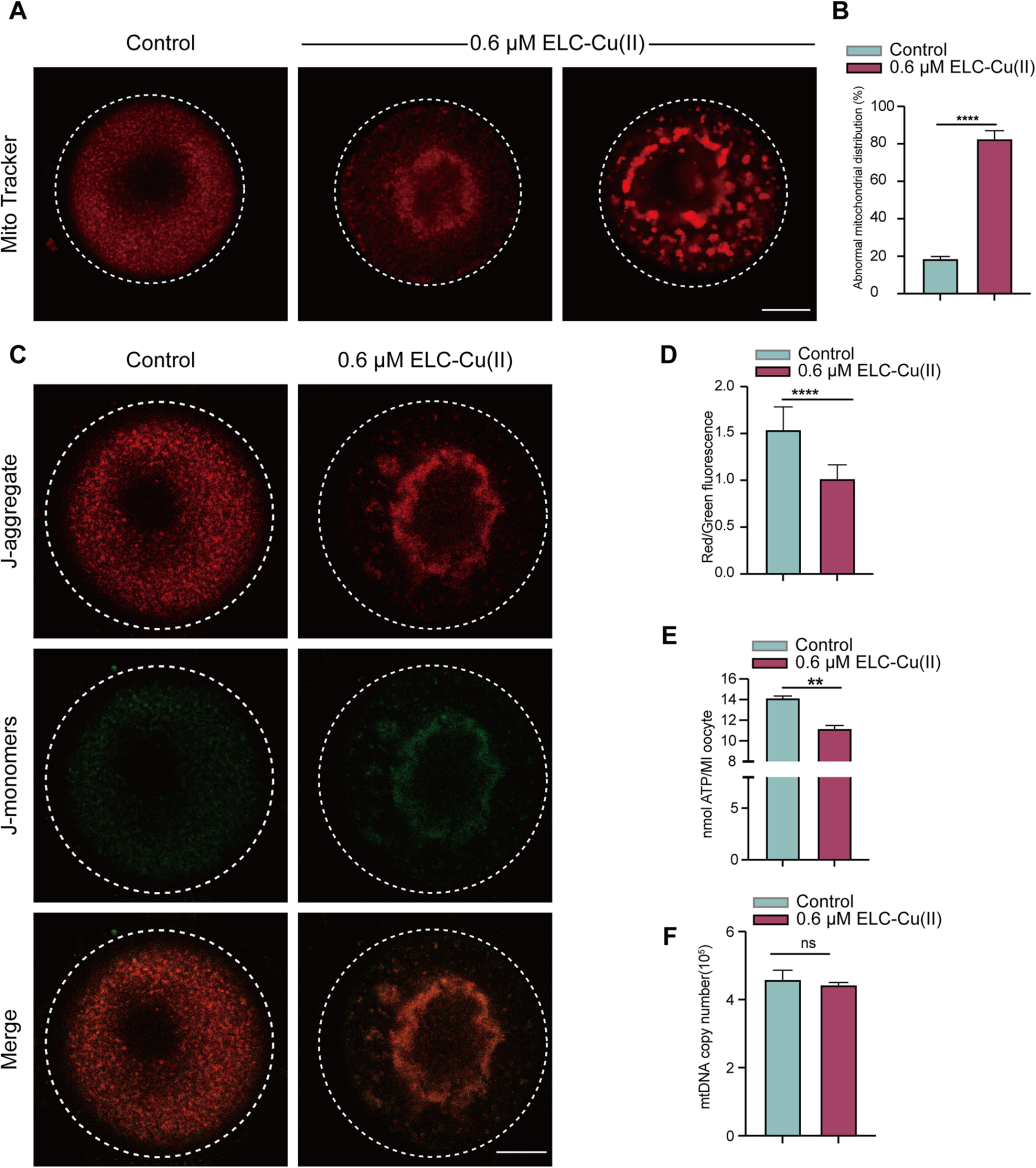
ELC-Cu(II) treatment disrupts mitochondrial distribution and function in mouse oocytes. **A** Representative fluorescence images of mitochondrial distribution in control and 0.6 μM ELC-Cu(II)-treated oocytes at MI stage. Scale bar =20 μm. **B** Quantification of the percentage of oocytes exhibiting abnormal mitochondrial distribution in the control (*n* = 72) and 0.6 μM ELC-Cu(II)-treated (*n* = 65) groups. **C** Representative fluorescence images of JC-1 staining to assess the mitochondrial membrane potential (ΔΨm) in control and 0.6 μM ELC-Cu(II)-treated oocytes. Red fluorescence indicates high mitochondrial membrane potential (J-aggregates), while green fluorescence indicates low potential (J-monomers). **D** Quantification of the red/green fluorescence intensity ratio from JC-1 staining in control (*n* = 36) and 0.6 μM ELC-Cu(II)-treated (*n* = 31) oocytes. **E** Intracellular ATP content in MI oocytes from control (*n* = 60) and 0.6 μM ELC-Cu(II)-treated (*n* = 60) group. **F** mtDNA copy numbers of GV oocytes in all three groups were analyzed by qPCR. Data represent the analysis of 10 GV oocytes for each treatment condition. Data in (**B**), (**D**), (**E**)and (**F**) are presented as the mean ± SEM. All results were derived from at least three independent experiments. **P < 0.01; ****P < 0.0001.

As mitochondria are primary targets of cuproptosis, we subsequently assessed the impact of ELC-Cu(II) on mitochondrial organization and bioenergetic function. In control oocytes, mitochondria displayed a relatively uniform distribution throughout the cytoplasm, as visualized by MitoTracker Red staining (Fig. 6A). In contrast, ELC-Cu(II)-treated oocytes exhibited abnormal mitochondrial clustering and aggregation, with 82.67 ± 2.53% of treated oocytes showing this phenotype compared to only 18.72 ± 0.69% in controls (*P* < 0.0001; Fig. 6B). To assess whether the altered mitochondrial distribution reflected functional impairment, we measured two key parameters of mitochondrial bioenergetics: membrane potential (ΔΨm) and ATP production. Mitochondrial membrane potential, assessed using the JC-1 fluorescent probe, was significantly reduced in ELC-Cu(II)-treated oocytes. The ratio of red fluorescence (J-aggregates, indicating high ΔΨm) to green fluorescence (J-monomers, indicating low ΔΨm) decreased from 1.540 ± 0.058 in controls to 1.017 ± 0.033 in treated oocytes (P < 0.0001; Fig. 6C, D). Consistent with impaired mitochondrial function, intracellular ATP levels were significantly reduced in ELC-Cu(II)-treated oocytes (11.01 ± 0.28 nM per MI oocyte) compared to controls (14.15 ± 0.13 nM per MI oocyte, P < 0.001; Fig. 6E). To determine whether the observed mitochondrial defects—encompassing abnormal distribution, dissipated membrane potential, and reduced ATP output—reflected a loss of mitochondrial mass rather than intrinsic functional impairment, we quantified relative mtDNA copy number as a surrogate measure of mitochondrial content by qPCR. The mtDNA copy number was comparable between control and ELC-Cu(II)-treated oocytes and did not differ significantly between the two groups (Fig. 6F), indicating that the mitochondrial dysfunction observed under cuproptotic stress arises from functional compromise rather than a reduction in mitochondrial quantity. Collectively, these findings demonstrate that ELC-Cu(II) treatment causes both structural and functional impairment of mitochondria without affecting their total mass, suggesting that the observed dysfunction primarily stems from direct disruption of the electron transport chain.

### ELC-Cu(II) triggers a predominantly post-transcriptional response in mouse oocytes

**Fig. 7:**
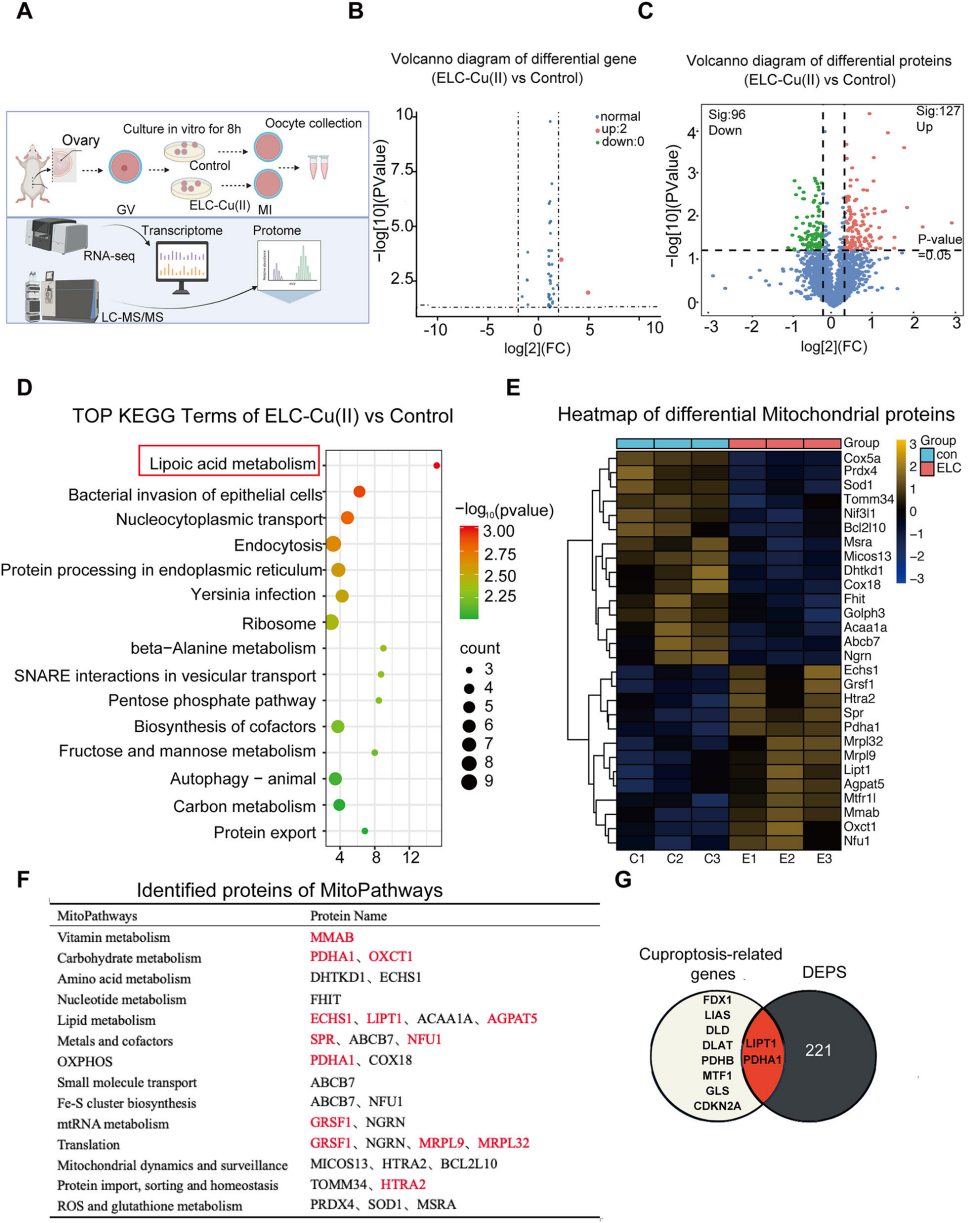
Multi-omics analysis reveals ELC-Cu(II)-induced molecular alterations in mouse oocytes. **A**Workflow for single-cell transcriptomic and proteomic analysis of oocytes from the control and 0.6 μM ELC-Cu(II)-treated oocytes at MI stage. Created with BioRender.com (https://BioRender.com/2ndil9l). **B**,**C** Volcano plots of DEGs (**B**) and DEPs (**C**) between the ELC-Cu(II)-treated and control oocytes. Upregulated genes are shown in red, and downregulated genes are shown in green. **D** KEGG term enrichment analysis of the identified DEPs, performed using Metascape. **E** Heatmap of 28 mitochondrial DEPs. **F** Altered proteins in key mitochondrial pathways (red, up; black, down). **G** Intersection of DEPs and cuprotosis-associated genes.

To elucidate the molecular mechanisms underlying ELC-Cu(II)-induced meiotic arrest in oocytes, we performed integrated single-cell transcriptomic and proteomic profiling of control and ELC-Cu(II)-treated oocytes at the metaphase I stage (Fig. 7A). Principal component analysis (PCA) revealed distinct clustering between the two groups at the proteomic level (Fig. S1E), whereas the transcriptomic profiles showed minimal separation (Fig. S1B), suggesting that the cellular response occurs predominantly through post-transcriptional regulation. Differential expression analysis confirmed this observation. Volcano plot analysis of transcriptomic data, using stringent criteria (|log₂FC| ≥ 2, FDR < 0.05), identified only 2 differentially expressed genes (DEGs; Fig. 7B). In striking contrast, proteomic volcano plot analysis identified 223 differentially expressed proteins (DEPs) using criteria of |log₂FC| ≥ 0.26 and adjusted P < 0.05 (Fig. 7C). This 112-fold difference between proteomic and transcriptomic changes highlights the oocyte’s reliance on post-transcriptional mechanisms to respond to cuproptotic stress. To identify the biological processes most affected by ELC-Cu(II) treatment, we performed KEGG pathway enrichment analysis on the 223 DEPs. The analysis revealed “lipoic acid metabolism” as the most significantly enriched pathway (Fig. 7D). Cross-referencing the DEPs with established cuproptosis-associated factors identified two key overlapping proteins: LIPT1 (lipoyltransferase 1) and PDHA1 (pyruvate dehydrogenase E1 alpha 1 subunit) (Fig. 7G), both of which are positive regulators of cuproptosis and were upregulated in ELC-Cu(II)-treated oocytes.

To investigate the mitochondrial-specific effects, we integrated our proteomic dataset with the MitoCarta 3.0 database, which identified 28 mitochondrial proteins among the DEPs. Hierarchical clustering analysis revealed distinct expression patterns between control and ELC-Cu(II)-treated groups (Fig. 7E). Of these 28 mitochondrial proteins, 13 were upregulated (including PDHA1, which is involved in oxidative phosphorylation), and 15 were downregulated. Notably, the downregulated proteins included ABCB7 (ATP-binding cassette sub-family B member 7) and NFU1 (NFU1 iron-sulfur cluster scaffold), both critical components of iron-sulfur cluster biosynthesis. Detailed pathway mapping of these altered mitochondrial proteins showed their involvement in key mitochondrial processes, including the TCA cycle, oxidative phosphorylation, lipid metabolism, and iron-sulfur cluster assembly (Fig. 7F). Quantitative analysis indicated that approximately 35.7% (10 of 28) of these mitochondrial proteins participate in core mitochondrial metabolic pathways. Together, these multi-omics analyses reveal that ELC-Cu(II) induces meiotic arrest through extensive proteomic remodeling rather than transcriptional reprogramming, with dysregulation of lipoic acid metabolism and iron-sulfur cluster biosynthesis as central molecular features.

### LIPT1 knockdown fails to rescue ELC-Cu(II)-induced meiotic arrest

Our proteomic data revealed upregulation of LIPT1 and PDHA1, two known positive regulators of cuproptosis, in ELC-Cu(II)-treated oocytes. LIPT1 functions as the rate-limiting mitochondrial lipoyltransferase that is responsible for protein lipoylation critical for TCA cycle function [16]. As cuproptosis is driven by copper-dependent aggregation of lipoylated proteins, we hypothesized that increased LIPT1 expression enhances protein lipoylation, thereby sensitizing oocytes to copper toxicity and precipitating meiotic arrest. Previous studies have established that oocytes lacking Pdha1 exhibit significant meiotic defects in vitro [17]; therefore, we focused our intervention on LIPT1.

To test whether LIPT1 upregulation contributes to the meiotic arrest, we performed RNA interference to knock down Lipt1 expression in oocytes. Microinjection of *Lipt1*-specific siRNA successfully reduced *Lipt1* mRNA levels to 0.28 ± 0.026-fold of control levels (*P* < 0.01; Fig. S2A), confirming effective knockdown. However, when LIPT1-depleted oocytes were subsequently treated with 0.6 μM ELC-Cu(II), the polar body extrusion rate remained severely impaired (4.2% ± 2.49% in the LIPT1-KD group vs. 12.49% ± 2.91% in the negative control siRNA group) (Fig. S2B, C). This lack of rescue indicates that LIPT1 knockdown alone was insufficient to prevent ELC-Cu(II)-induced meiotic arrest under these experimental conditions.

### NAD^+^ precursor supplementation partially rescues ELC-Cu(II)-induced meiotic defects

**Fig. 8:**
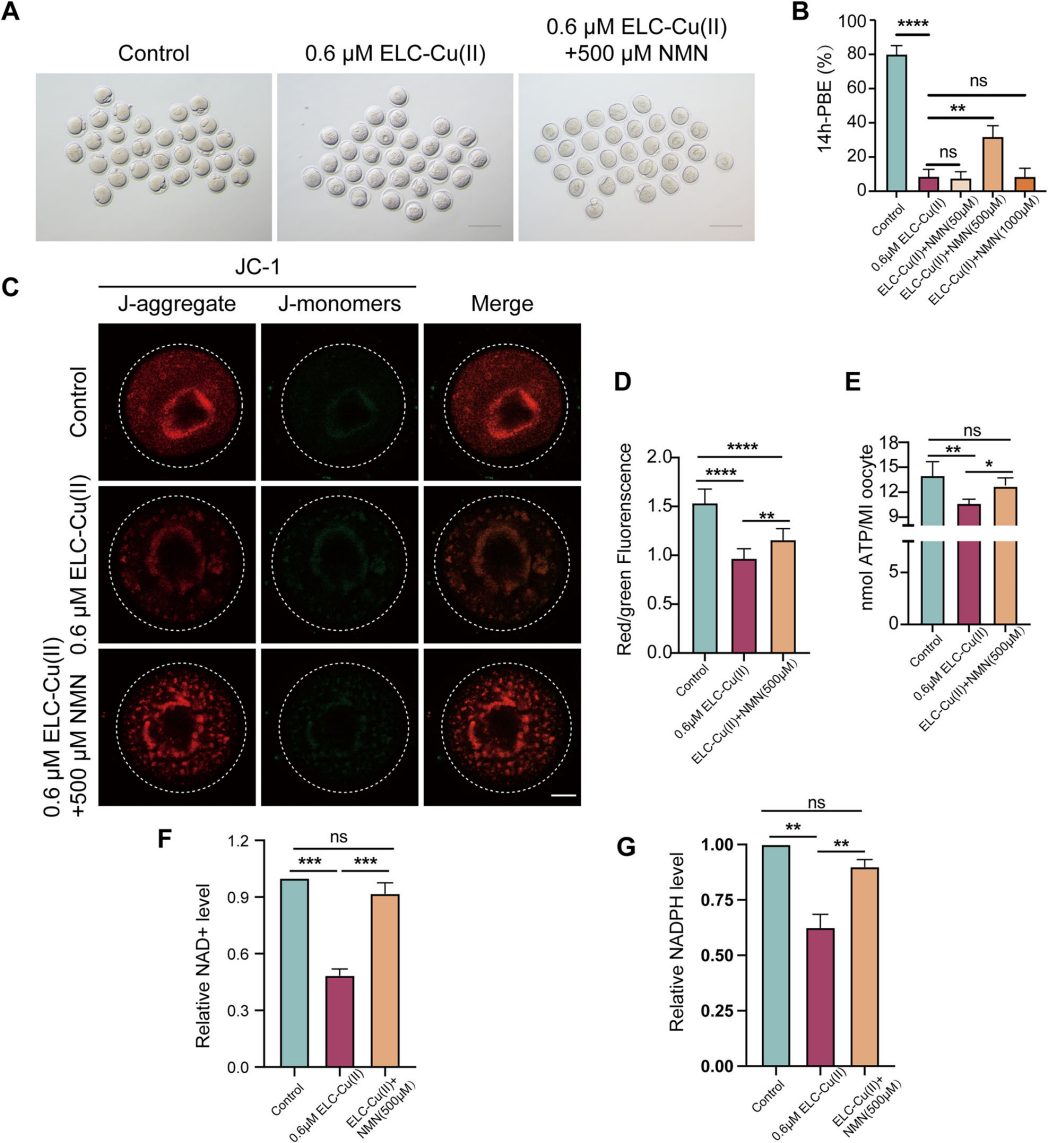
NMN supplementation rescues ELC-Cu(II)-induced meiotic defects and mitochondrial dysfunction. **A** Representative images of MII oocytes from the control, 0.6 μM ELC-Cu(II), and 0.6 μM ELC-Cu(II) + 500 μM NMN co-treatment groups, observed at 14 h after release from IBMX. Scale bar, 100 μm. **B** Quantification of the first polar body extrusion (PBE) rate in the control (*n* = 88), ELC-Cu(II)-treated (*n* = 93), and ELC-Cu(II) + NMN co-treated (50 μM *n* = 90; 500 μM *n* = 95; 1000 μM *n* = 105) groups. **C** Representative fluorescence images of JC-1 staining in oocytes from the three experimental groups at the MI stage. **D** Quantification of the red/green fluorescence intensity ratio from JC-1 staining in the control (*n* = 27), ELC-Cu(II)-treated (*n* = 35), and ELC-Cu(II) + 500 μM NMN (*n* = 27) groups. **E** Measurement of relative ATP levels in oocytes from the control (*n* = 60), ELC-Cu(II)-treated (*n* = 60), and ELC-Cu(II) + 500 μM NMN (*n* = 60) groups at the MI stage. Measurement of intracellular NAD+ (**F**) and NADPH (**G**) levels in MI oocytes across the control(n = 150), ELC-Cu(II) (*n* = 150), and ELC-Cu(II) + NMN (n = 150) groups. Data in (**B**), (**D**), (**E**), (**F**)and (**G**) are presented as the mean ± SEM. All results were derived from at least three independent experiments. *P <0.05; **P < 0.01; ***P <0.001;****P < 0.0001.

Given that LIPT1 knockdown failed to prevent meiotic arrest, we considered alternative therapeutic strategies targeting upstream metabolic pathways. The lipoylation pathway is regulated by NAD^+^-dependent delipoylases, particularly Sirt2 [18] and Sirt4 [19], which remove lipoyl modifications from target proteins. Furthermore, NAD^+^ serves as a substrate for NAD^+^ kinase (NADK) to generate NADP^+^, which is subsequently reduced to NADPH—a critical reducing equivalent for the biogenesis of iron-sulfur (Fe-S) clusters [20]. Our previous proteomic analysis identified the Fe-S cluster assembly pathway as being significantly compromised under cuproptotic stress. Based on recent evidence that nicotinamide mononucleotide (NMN), a primary NAD^+^ precursor, mitigates cuproptosis in somatic cells by elevating intracellular Sirt2 and NADPH levels [21], we tested whether NMN supplementation could protect oocytes from ELC-Cu(II)-induced damage. Co-treatment of oocytes with 500 μM NMN and 0.6 μM ELC-Cu(II) significantly improved meiotic maturation compared to ELC-Cu(II) treatment alone. The polar body extrusion rate increased from 8.6 ± 2.42% (ELC-Cu(II) alone, *n* = 93) to 31.89 ± 3.70% (ELC-Cu(II) + NMN, *n* = 95; *P* < 0.001), representing a 3.7-fold improvement (Fig. 8A, B). In contrast, neither a lower (50 μM) nor a higher (1000 μM) concentration of NMN conferred any improvement in meiotic maturation, yielding PBE rates of 8.6 ± 2.42% (n = 90) and 8.5 ± 2.81% (*n* = 92), respectively, showing no significant difference from the ELC-Cu(II) treatment group. However, this rescue was partial, as the PBE rate remained significantly lower than thatof the control group (80.06 ± 2.94%, n = 88; *P* < 0.0001). To determine whether the improved meiotic outcomes reflected restored mitochondrial function, we assessed mitochondrial bioenergetics in NMN-treated oocytes. JC-1 staining revealed that NMN co-treatment substantially restored mitochondrial membrane potential, with the red/green fluorescence ratio increasing from 0.969 ± 0.031 in the ELC-Cu(II) group to 1.159 ± 0.034 in the ELC-Cu(II) + NMN group (*P* < 0.001; Fig. 8C, D). Consistently, intracellular ATP levels were partially restored from 10.64 ± 0.267 nM per MI oocyte (ELC-Cu(II) alone) to 12.69 ± 0.311 nM per MI oocyte (ELC-Cu(II) + NMN; *P* < 0.01; Fig. 8E), although they remained below control levels. Given the pivotal role of NADPH in Fe-S cluster biogenesis, we further investigated whether the protective effect of NMN against cuproptosis-induced meiotic arrest is mediated, at least in part, through restoration of intracellular NADPH availability. In support of this notion, ELC-Cu(II) treatment caused a marked depletion of both NAD⁺ and NADPH (Fig. 8F, G), consistent with the broad metabolic disruption induced by cuproptotic stress. Notably, co-treatment with the NAD⁺ precursor NMN significantly restored the intracellular levels of both metabolites (Fig. 8F, G), confirming that NMN effectively replenishes the NAD(P)H pool under cuproptotic conditions.

These findings demonstrate that supplementation with the NAD+ precursor NMN provides significant protection against ELC-Cu(II)-induced meiotic arrest, likely by restoring mitochondrial bioenergetic function. The partial nature of the rescue suggests that while NAD+ metabolism is a critical determinant of oocyte resilience to cuproptosis, additional damage pathways may require concurrent therapeutic intervention.

## Discussion

This study provides the first comprehensive characterization of cuproptosis in mammalian oocytes and identifies this copper-dependent cell death pathway as a mechanism of meiotic failure. We demonstrate that pharmacological induction of cuproptosis with ELC-Cu(II) causes metaphase I arrest through a cascade of cellular defects, including spindle disorganization, chromosome misalignment, defective kinetochore-microtubule attachments, and sustained spindle assembly checkpoint activation. Mechanistically, ELC-Cu(II) treatment triggers the canonical hallmarks of cuproptosis—intracellular copper accumulation, FDX1 downregulation, and proteotoxic stress [10] —accompanied by mitochondrial dysfunction characterized by reduced membrane potential and ATP depletion. Importantly, integrated transcriptomic and proteomic analyses reveal that the oocyte’s response to cuproptotic stress is overwhelmingly post-transcriptional, with 223 differentially expressed proteins but only two differentially expressed genes. While targeted depletion of the lipoyltransferase LIPT1 failed to prevent meiotic arrest, supplementation with the NAD+ precursor NMN partially restored both polar body extrusion and mitochondrial function. These findings establish cuproptosis as a previously unrecognized threat to oocyte developmental competence and highlight NAD+ metabolism as a potential therapeutic target.

### Post-transcriptional regulation dominates the oocyte cuproptosis response

A striking finding of our study is the extreme disparity between transcriptomic stability and proteomic remodeling in response to ELC-Cu(II) treatment. The 112-fold difference (223 DEPs versus 2 DEGs) represents one of the most pronounced examples of post-transcriptional regulation documented in oocytes under acute stress. This observation aligns with the well-established principle that mammalian oocytes are transcriptionally quiescent during meiotic maturation and rely exclusively on translational control, protein degradation, and post-translational modifications to regulate cellular processes [1]. Our findings extend this concept to stress responses, demonstrating that even when confronted with a potent cytotoxic insult, oocytes do not mount a transcriptional response but instead reshape their proteome through post-transcriptional mechanisms. The proteomic signature of cuproptosis in oocytes centers on dysregulation of lipoic acid metabolism and iron-sulfur cluster biosynthesis, consistent with the established molecular basis of this cell death pathway in cancer cells [15, 22]. The upregulation of LIPT1 and PDHA1, both positive regulators of cuproptosis [8, 23], suggests that ELC-Cu(II) treatment enhances the lipoylation of TCA cycle enzymes, thereby increasing their susceptibility to copper-induced aggregation. Conversely, the downregulation of Fe-S cluster assembly factors (ABCB7, NFU1) indicates disruption of this essential mitochondrial process [24, 25], which is both a consequence of cuproptosis [8] and a contributor to mitochondrial dysfunction. This dual dysregulation—increased substrate availability for copper toxicity (lipoylated proteins) and impaired repair capacity (Fe-S cluster assembly)—likely creates a feed-forward cycle that amplifies cellular damage.

### LIPT1 inhibition is insufficient to reverse cuproptosis-induced meiotic arrest

Despite the central role of protein lipoylation in cuproptosis [26], targeted knockdown of LIPT1 failed to prevent ELC-Cu(II)-induced meiotic arrest. This negative result is informative and suggests several non-mutually exclusive possibilities. First, the achieved knockdown efficiency ( ~ 72% reduction in mRNA) may be insufficient to substantially reduce LIPT1 protein levels or enzymatic activity, particularly given that oocytes contain abundant maternally inherited proteins that turn over slowly. Second, compensatory mechanisms may exist; other lipoyltransferases or alternative lipoylation pathways could maintain sufficient protein lipoylation to sensitize cells to copper toxicity even when LIPT1 is depleted [27]. Third, and most likely, once cuproptotic stress is initiated, the resulting cellular damage—including mitochondrial dysfunction, proteotoxic stress, and spindle defects—may trigger irreversible cascades that cannot be prevented by blocking the initial sensitizing step. The failure of LIPT1 knockdown highlights a critical distinction between understanding a pathway and therapeutically intervening in it. While LIPT1-mediated lipoylation is necessary for cuproptosis sensitivity in cancer cells [28], blocking this single node is inadequate in oocytes. This contrasts with the situation in proliferating cancer cells, where LIPT1 or DLAT depletion can confer resistance to cuproptosis inducers [28–30]. The difference may reflect the oocyte’s limited capacity for protein synthesis and repair during meiotic maturation [31], such that once damage accumulates, the cell cannot recover even if the initiating insult is removed.

### NAD+ supplementation provides partial protection through metabolic restoration

In contrast to LIPT1 knockdown, NMN supplementation significantly improved meiotic outcomes and mitochondrial function in ELC-Cu(II)-treated oocytes. The protective effect of NMN likely operates through multiple mechanisms. First, NMN increases intracellular NAD+ levels, which activate NAD^+^-dependent delipoylases such as Sirt2 [18] and Sirt4 [19, 32]. These enzymes remove lipoyl modifications from TCA cycle proteins, potentially reducing the pool of available substrates for copper-induced aggregation. Second, NAD^+^ is a precursor for NADPH synthesis, and NADPH is essential for both FDX1 function [11] and iron-sulfur cluster biosynthesis [33]. By replenishing the NADPH pool, NMN may partially restore Fe-S cluster assembly, thereby mitigating one of the key downstream consequences of cuproptosis. Third, NAD^+^ is required for mitochondrial ATP production and maintenance of cellular redox homeostasis [34]; NMN supplementation may therefore enhance overall mitochondrial resilience to cuproptotic stress. The partial nature of the NMN rescue is significant. While polar body extrusion improved 3.7-fold, it remained far below control levels, and mitochondrial parameters showed similar incomplete restoration. This incomplete rescue raises a critical question regarding the nature of the observed MI arrest: whether it represents a reversible physiological pause or an irreversible commitment to cell death. Given the severity of the mitochondrial collapse, ATP depletion, and proteotoxic stress arising from protein aggregation, we propose that ELC-Cu(II) treatment initiates a “pre-lethal” metabolic crisis that progressively erodes oocyte viability. Within this framework, NMN may bolster metabolic resilience in oocytes that have not yet crossed a critical damage threshold; however, once the cuproptosis pathway is fully executed, NMN appears insufficient to reverse the ensuing structural damage to the spindle apparatus or to override the terminal signaling sustaining persistent SAC activation. Collectively, these considerations suggest that the observed MI arrest more likely represents an irreversible manifestation of incipient cell death than a recoverable meiotic delay, underscoring the importance of early intervention before irreversible cellular damage accumulates. Nonetheless, the clear protective effect of NMN demonstrates that NAD+ metabolism is a critical determinant of oocyte resilience to cuproptotic stress and identifies this pathway as a tractable therapeutic target. This finding is consistent with recent evidence that NMN partially rescues cuproptosis in somatic cells by upregulating Sirt2 and increasing intracellular NADPH levels [21].

### Cuproptosis and oocyte quality: physiological and clinical implications

While our study employed a pharmacological inducer of cuproptosis, the findings raise important questions about the physiological relevance of this pathway to oocyte biology and female fertility. Copper homeostasis is tightly regulated, and dysregulation of copper metabolism has been implicated in several reproductive disorders. Elevated copper levels have been reported in women with polycystic ovary syndrome (PCOS) [35, 36] and endometriosis [37], two common causes of infertility. Moreover, environmental and occupational copper exposure remains a concern in certain populations. Our findings suggest that copper-induced toxicity in oocytes may operate through the cuproptosis pathway, providing a mechanistic framework for understanding how copper dysregulation impairs oocyte quality. The mechanisms we have identified—mitochondrial dysfunction, proteotoxic stress, and disrupted Fe-S cluster biosynthesis—are also implicated in oocyte aging [38, 39]. Aged oocytes exhibit declining mitochondrial function, increased protein oxidation and aggregation [40], and impaired spindle assembly [41]. It is tempting to speculate that low-level, chronic cuproptotic stress could contribute to the age-related decline in oocyte quality, although this hypothesis requires direct experimental testing. Furthermore, our identification of NAD^+^ metabolism as a protective factor aligns with growing evidence that NAD^+^ decline is a hallmark of aging and that NAD^+^ precursor supplementation may ameliorate age-related functional decline in multiple tissues [42, 43], including oocytes [44, 45].

### Comparison with other forms of regulated cell death in oocytes

Cuproptosis joins a growing list of regulated cell death pathways characterized in oocytes, including apoptosis [46, 47], ferroptosis [7, 48], autophagy [49, 50], and necroptosis [51]. Each pathway appears to be triggered by distinct stressors and operates through specific molecular mechanisms, yet they share common downstream consequences—mitochondrial dysfunction, proteostasis collapse, and meiotic failure. What distinguishes cuproptosis from these other pathways is its unique dependence on metal-protein interactions and the specific targeting of lipoylated metabolic enzymes [8]. Unlike ferroptosis, which involves lipid peroxidation driven by iron [52], cuproptosis involves protein aggregation driven by copper [8]. Unlike apoptosis, which is initiated by extrinsic or intrinsic apoptotic signals and executed by caspases [53], cuproptosis appears to be caspase-independent and mechanistically distinct [10]. An important question is whether cuproptosis represents a true “cell death” pathway in oocytes or rather a form of functional impairment that arrests meiosis without killing the cell. Our study focused on meiotic progression rather than cell viability per se, and the distinction is not merely semantic. If oocytes arrested by cuproptosis retain viability but are unable to complete maturation, this suggests a potentially reversible state that might be amenable to intervention. Conversely, if cuproptosis triggers irreversible cell death pathways, therapeutic strategies would need to focus on prevention rather than rescue. Future studies using live-cell imaging and viability assays will be needed to resolve this question.

### Limitations and future directions

Several limitations of this study warrant consideration. First, we relied on pharmacological induction of cuproptosis using ELC-Cu(II), which delivers supraphysiological copper loads. While this approach clearly demonstrates that oocytes are vulnerable to cuproptosis, it remains to be determined whether endogenous copper dysregulation under physiological or pathological conditions induces similar effects. Second, our mechanistic studies were conducted exclusively in mouse oocytes; validation in human oocytes will be essential to assess clinical relevance, although practical and ethical constraints limit such studies. Third, while we identified NAD^+^ metabolism as a potential therapeutic target, the optimal timing, dosage, and delivery method for NAD^+^ precursors remain to be established [54]. Fourth, our proteomic analysis provided a snapshot at a single time point (metaphase I); time-resolved multi-omics would better capture the dynamics of the cuproptosis response. Furthermore, the inherently limited cellular input of oocyte-based studies—substantially lower than that achievable with bulk tissue samples—poses a challenge to mass spectrometric sensitivity. As a result, low-abundance signaling proteins and rare transcription factors may fall below the reliable detection threshold, potentially introducing a systematic bias toward the identification of more abundant proteins in our proteomic dataset. Future studies should address several key questions: What are the endogenous triggers of cuproptosis in oocytes, if any? Does copper dysregulation in conditions such as PCOS or Wilson disease increase the risk of cuproptosis? Can cuproptosis markers predict oocyte quality in assisted reproductive technology settings [55]? Does age-related decline in NAD^+^ levels [56] sensitize oocytes to cuproptosis? Can combinatorial interventions—such as NMN plus antioxidants or chaperone inducers—achieve more complete rescue? Addressing these questions will clarify the physiological and clinical significance of cuproptosis in reproductive biology.

In summary, we have established cuproptosis as a mechanism of meiotic failure in mammalian oocytes, characterized the cellular and molecular basis of this phenomenon, and identified NAD+ metabolism as a determinant of oocyte resilience to copper-induced stress. Our findings reveal that oocytes respond to cuproptotic stress through extensive post-transcriptional remodeling rather than transcriptional reprogramming, highlighting their unique biology [3]. While targeted inhibition of protein lipoylation was ineffective, metabolic support through NAD^+^ precursor supplementation provided partial protection [21], suggesting that supporting cellular metabolism may be more effective than blocking specific damage pathways. These results advance our understanding of oocyte quality control mechanisms and identify potential therapeutic avenues for protecting oocytes from metal toxicity and metabolic stress.

## Materials and methods

### Mice

Female ICR mice (3-6 weeks old) were housed under standardized housing conditions with controlled temperature (20–23 °C) and a 12-hour light/dark cycle. Mice were provided with ad libitum access to standard rodent chow and water. All animal procedures were performed in accordance with the guidelines set by the Animal Research Committee of Guangdong Second Provincial General Hospital and were approved by the committee (Ethical Approval No. 2022-DW-KZ-103-01).

### Antibodies and reagents

Mouse monoclonal anti–α-tubulin–fluorescein isothiocyanate (FITC) antibody was purchased from Sigma-Aldrich (St. Louis, MO, USA; catalog no.: F2168); rabbit polyclonal anti-Mad2L1 antibody was purchased from Proteintech (catalog no.: 10337-1-AP); human anti-centromere antibody was purchased from Antibodies Incorporated (Davis, CA, USA; catalog no.: A9657); anti-γ-tubulin antibody was purchased from Abclonal (catalog no.: 60008-1-Ig); FDX1 antibody was purchased from Pro-teintech (catalog no.: 12592-1-AP); 3-isobutyl-1-metyl-xanthine (IBMX) was purchased from Sigma-Aldrich (catalog no.: I5879); Cu(II)-Elesclomol(ELC-Cu(II) was purchased from MedChemExpress (cata- log no.: HY-156376). β-Nicotinamide mononucleotide (NMN) was purchased from Glpbio (catalog no.: GC16971)

### Oocytes collection and in vitro maturation

Germinal vesicle (GV)-stage oocytes were collected from ovaries of euthanized female ICR mice. Ovaries were dissected and placed in M2 medium (Sigma-Aldrich) supplemented with 200 μM 3-isobutyl-1-methylxanthine (IBMX; Sigma-Aldrich, I5879) to maintain meiotic arrest. and subsequently cultured in vitro in M16 medium (Sigma-Aldrich) under mineral oil at 37 °C with 5% CO₂. At designated time points, cultured oocytes were harvested for analysis.

### Oocytes treatment

Oocytes were cultured in M16 medium with 0-1 μM ELC-Cu(II) following random assignment to assess the effect on first polar body extrusion. The compound was diluted from a DMSO stock into M16 medium, with the final DMSO concentration maintained at 0.3% for all conditions. A submaximal inhibitory concentration of 0.6 μM was identified in this initial screen and was therefore used in all subsequent experiments.

### Immunofluorescence staining

Oocytes were fixed in 4% paraformaldehyde (PFA) in PBS for 30 min at room temperature, then permeabilized with 0.5% Triton X-100 for 20 min at room temperature. To block nonspecific binding, samples were incubated in PBS containing 1% BSA for 1 h. Oocytes were then incubated overnight at 4 °C with primary antibodies: FITC-conjugated anti-α-tubulin (1:800; Sigma-Aldrich, F2168), anti-γ-tubulin (1:500; Abclonal, A9657), or anti-ACA (1:200; Antibodies Incorporated, 15-234-0001).

After three washes in PBST (PBS containing 0.1% Tween-20), oocytes were incubated with appropriate Alexa Fluor-conjugated secondary antibodies for 1 h at room temperature. DNA was then counterstained with Hoechst 33342 for 10 min. Finally, the samples were mounted on glass slides with an anti-fade medium and imaged using a Zeiss LSM 900 laser scanning confocal microscope. Fluorescence intensity was quantified as previously described [57].

### Chromosome spreading

Oocytes were briefly treated with acidic Tyrode’s buffer (pH 2.5) at 37 °C for 30 sec to remove the zona pellucida. then recovered in M2 medium for 10 min. Zona-free oocytes were transferred to a droplet of 1% paraformaldehyde (PFA) containing 0.15% Triton X-100 on a glass slide and air dried. Chromosomes were counterstained with Hoechst33342 and examined by confocal microscopy.

### ATP measurement

Intracellular ATP levels were measured using an Enhanced ATP Assay Kit (Beyotime, S0027) according to the manufacturer’s protocol. For each replicate, 20 oocytes were collected in 200 μL tubes and stored at -80 °C until analysis. A standard curve was generated using ATP concentrations ranging from 0 to 1 μM. Luminescence was measured on a TECAN Spark microplate reader, and ATP content was calculated from the standard curve.

### Cellular copper measurement

Intracellular copper (Cu²⁺) concentrations were quantified using a commercial Copper Colorimetric Assay Kit (Cat. No. E-BC-K775-M; Elabscience) in accordance with the manufacturer’s protocol. For each replicate, 200 oocytes were homogenized to prepare the sample lysates. A standard curve was generated using known Cu²⁺ concentrations ranging from 0 to 5 μM. Sample absorbance was measured with a TECAN Spark microplate reader, and the corresponding Cu²⁺ concentration for each sample was determined by interpolation from the standard curve.

### Mitochondrial staining and functional assays

For mitochondrial distribution, oocytes were incubated with MitoTracker Red CMXRos (200 nM; Beyotime, C1035) in M2 medium for 30 min at 37 °C, washed three times in PBS, and immediately imaged.

Mitochondrial membrane potential (ΔΨm) was assessed using the JC-1 probe (Beyotime, C2003S). Oocytes were loaded with JC-1 (10 μg/mL) for 30 min at 37 °C, washed, and imaged. The ratio of red (J-aggregates) to green (J-monomers) fluorescence was calculated to quantify ΔΨm.

### Quantification of mitochondrial DNA (mtDNA) copy number in oocytes

Mitochondrial DNA copy number per oocyte was quantified by real-time quantitative PCR (qPCR) as previously described [58]. Amplification was performed using mouse-specific mtDNA primers (mtB6): forward, 5′- AACCTGGCACTGAGTCACCA-3′; reverse, 5′-GGGTCTGAGTGTATATATCATGAAGAGAAT-3′ [59]. An absolute quantification standard curve was constructed from 10-fold serial dilutions of a purified plasmid DNA template spanning 10³ to 10⁸ copies/μL, and amplification efficiency was verified using the Roche qPCR efficiency calculator. For sample preparation, individual oocytes were lysed in 10 μL of lysis buffer containing 0.12 mg/mL Proteinase K at 55 °C for 2 h, followed by enzyme inactivation at 95 °C for 10 min. A 2 μL aliquot of each lysate was used as the qPCR template. Thermal cycling comprised an initial denaturation at 95 °C for 10 min, followed by 40 cycles of denaturation (95 °C, 15 s) and combined annealing/extension (60 °C, 1 min), with a final extension at 72 °C for 1 min. Standard curves consistently achieved correlation coefficients (R²) > 0.97 across the entire quantification range (10³–10⁸ copies). All reactions were performed in technical triplicate, and a minimum of ten germinal vesicle (GV)-stage oocytes collected from three independent mice were analyzed per condition.

### Measurement of NAD(H) and NADP(H) levels

Intracellular NAD⁺ and NADPH concentrations were quantified using commercially available colorimetric assay kits (cat. nos. S0176S and S0180S; Beyotime, Shanghai, China) in strict accordance with the manufacturer’s instructions. Following the indicated treatments, oocytes were washed three times in ice-cold PBS supplemented with polyvinyl alcohol (PVA-PBS) and subsequently lysed in 50 μL of the provided extraction buffer. For NAD⁺ quantification, 20 μL of the resulting lysate was transferred to a 96-well plate and mixed with an ethanol dehydrogenase working solution. For NADPH quantification, 50 μL of the lysate was combined with a glucose-6-phosphate dehydrogenase (G6PD) working solution. In both assays, samples were incubated at 37 °C for 10 min to allow the enzymatic reaction to proceed, after which a chromogenic detection solution was added to each well and incubated for a further 30 min at 37 °C. Absorbance was measured at 450 nm using a SPARK multimode microplate reader (TECAN), and analyte concentrations were interpolated from a standard curve prepared in parallel. All measurements were conducted across at least three independent biological replicates.

### Protein analysis using the Wes-Protein simple system

The expression of FDX1 and β-tubulin was evaluated using the Jess™ automated capillary electrophoresis system (ProteinSimple, USA) according to the manufacturer’s protocol. The following primary rabbit antibodies were used: anti-FDX1 (1:50, ProteinTech, Cat# 12592-1-AP) and anti-β-tubulin (1:50, Cell Signaling Technology).

### Microinjection and post-injection oocyte culture

In vitro-transcribed cRNAs were diluted in RNase-free water to working concentrations of 20 ng/μL (H2B-mCherry) and 500 ng/μL (cyclin B1-GFP). GV-stage oocytes were collected and transferred into 20 μL droplets of M2 medium supplemented with 200 μM IBMX on a culture dish lid, which was then covered with mineral oil to prevent evaporation. Microinjections were carried out on an inverted microscope (Nikon) equipped with motorized micromanipulators and a FemtoJet 4i microinjector (Eppendorf), using holding and injection pipettes fabricated with a pipette puller and a microforge. To maximize cell viability, each injection batch was completed within 20 min. Following injection, oocytes were allowed to recover in M2-IBMX medium for 10 min and subsequently cultured for an additional 3 h to permit adequate cRNA translation and reporter protein expression. Meiotic arrest was then released by transferring oocytes to IBMX-free M16 medium for the live-imaging experiments described in Fig. 2C.

### Single-cell RNA-seq library construction

RNA-seq libraries were generated from MI oocytes of the Control and ELC-Cu(II)-treated groups following the Smart-seq2 protocol [60]. Prior to library preparation, oocytes were washed three times in PBS containing 0.2% BSA and collected in a lysis buffer supplemented with an RNase inhibitor. For data analysis, sequencing reads were aligned to the mouse reference genome (mm9) using HISAT2 (v2.1.0). Gene-level read counts were obtained with FeatureCounts (v2.0.1) based on the Gencode mm9 annotation. Subsequently, differential expression analysis was performed using the DESeq2 package. The analysis included an internal estimation of size factors for read count normalization prior to differential testing.

### LC–MS/MS analysis of mouse oocytes

To generate a spectral library for subsequent data-independent acquisition (DIA) analysis, Experiments were performed using three biological replicates per group, each composed of 29 MI oocytes from which the zona pellucida was removed by hyaluronidase digestion. Oocytes per sample were lysed in 50 μL of 0.2% sodium deoxycholate on ice for 30 min. Following lysis, proteins were reduced with 5 mM DL-dithiothreitol (Sigma, D9779) and alkylated with 10 mM iodoacetamide (Sigma, I1149). Protein digestion was carried out overnight with a Trypsin/Lys-C Mix (Promega, V5071). The resulting peptides were desalted on SOLAμ HRP SPE spin plates (Thermo Scientific, 60209-001), and the eluent was vacuum-dried. Dried peptides were redissolved in 0.1% formic acid for LC-MS/MS analysis. LC-MS/MS was conducted on a timsTOF Pro mass spectrometer (Bruker Daltonics) operating in DIA mode. Peptides were separated over a 120-min non-linear gradient. Mass spectrometry data were acquired over an m/z range of 300-1500 and a1/K0 range of 0.7-1.3 V·s cm⁻², with a cycle time of 1.23 s (one MS1 scan followed by ten PASEF MS/MS scans).

Spectral data were searched against the Mus musculus UniProt database (UP000000589) using DIA-NN, with precursor and fragment mass tolerances set to 15 ppm and 0.02 Da, respectively

### Declaration of generative AI

During the preparation of this work the author(s) used Gemini-2.5 Pro in order to improve language. After using this tool/service, the author(s) reviewed and edited the content as needed and take(s) full responsibility for the content of the published article.

### Statistical analysis

All data were obtained from at least three independent experiments and are presented as mean ± SEM. The sample size for each group is indicated as (n) in the corresponding figures or legends. Statistical analyses were performed using GraphPad Prism 8. For comparisons between two independent groups, a two-tailed unpaired Student’s *t*-test was employed. For experiments involving three or more groups, differences were assessed by one-way analysis of variance (ANOVA) followed by Tukey’s honestly significant difference (HSD) post hoc test for multiple comparisons. Statistical significance was defined as *P* < 0.05.

## Supplementary information

Figure legends.

Supplementary Figure 1.

Supplementary Figure 2.

dataset1

dataset3

dataset2

dataset4

## Data Availability

The raw sequence data reported in this paper have been deposited in the China National Center for Bioinformation / Genome Sequence Archive which are publicly accessible at https://ngdc.cncb.ac.cn/gsa/s/T3p5S27X.
