# Supplementary material for: Cuproptosis causes meiotic metaphase I arrest by disrupting mitochondrial functions in oocytes
Source: Cell Death Discov. 2026 May 23;12:329. doi: 10.1038/s41420-026-03168-x (PMC13448713; doi:10.1038/s41420-026-03168-x)
Supplement: Supplementary file 1 [file 41420_2026_3168_MOESM7_ESM.pdf]

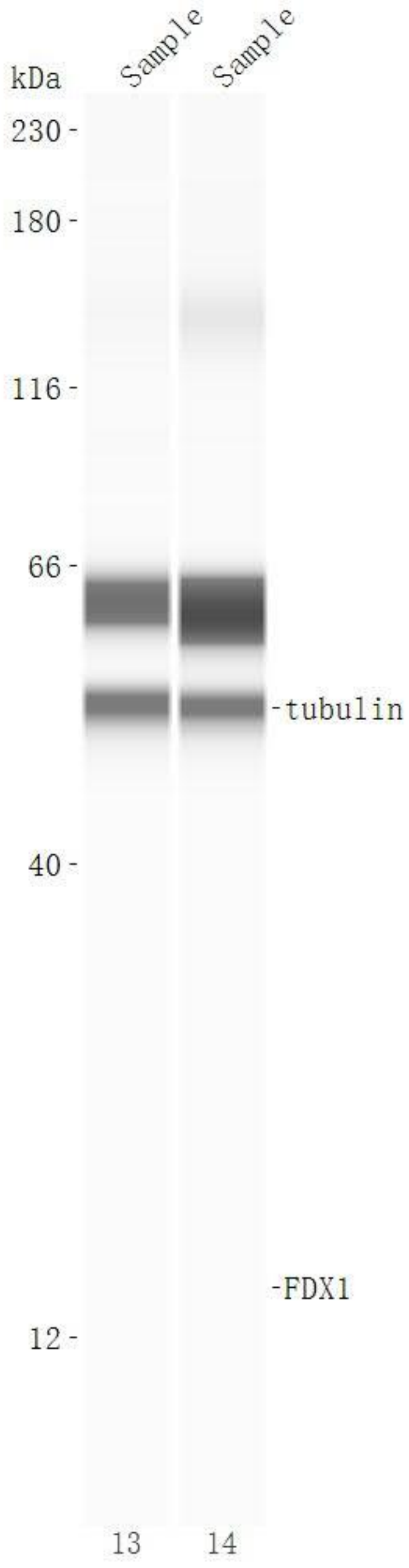

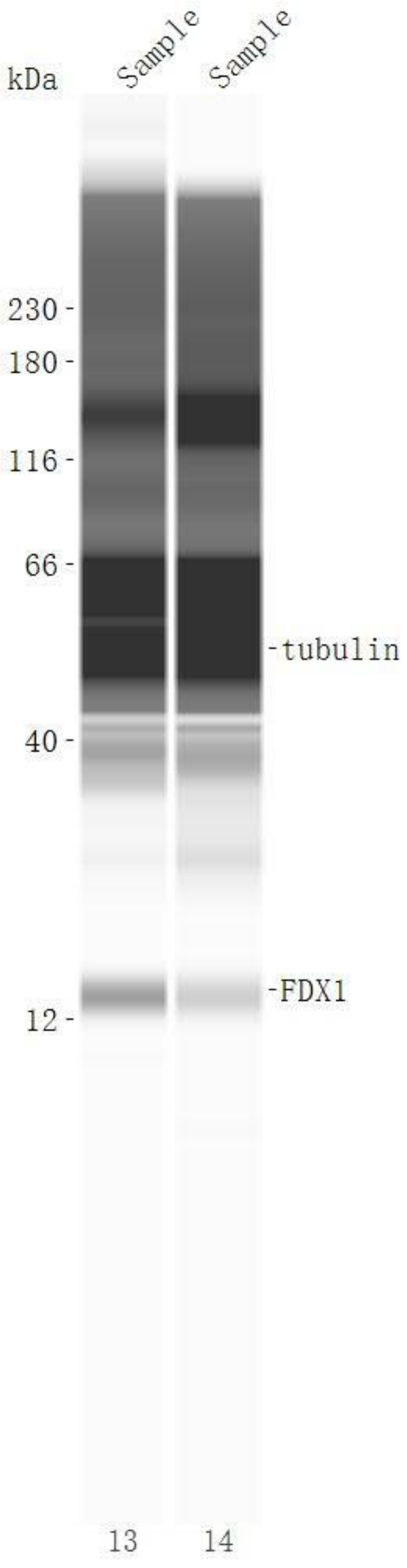

Sample	Primary	Capillary	FDX1	tubulin
Sample	Primary	13(Control)	58397.8	1301468.0
Sample	Primary	14(ELC-Cu(II)	34076.3	1228786.0
