# Supplementary material for: Cuproptosis causes meiotic metaphase I arrest by disrupting mitochondrial functions in oocytes
Source: Cell Death Discov. 2026 May 23;12:329. doi: 10.1038/s41420-026-03168-x (PMC13448713; doi:10.1038/s41420-026-03168-x)
Supplement: Supplementary file 2 [file 41420_2026_3168_MOESM1_ESM.docx]

**Figure legends**

## **Supplementary Figure 1.** **Global Transcriptomic and Proteomic Profiling of Single Oocytes Following ELC-Cu(II) Treatment.**

(A) Total mRNA expression levels (represented by total read counts) in control and ELC-Cu(II)-treated oocytes. Data are presented as mean ± SEM (n = 3 biological replicates per group).(B) Principal Component Analysis (PCA) of single-oocyte transcriptomes showing distinct clustering of the control and ELC-Cu(II)-treated groups (n = 3 per group).(C) Venn diagrams illustrating the overlap of identified protein among three biological replicates for both control and ELC-Cu(II) groups.(D) Pearson correlation coefficients (r) between samples. (E) PCA of single-oocyte proteomes showing a clear separation between control and ELC-Cu(II)-treated groups (n = 3 per group).

## **Supplementary Figure 2. The role of LIPT1 in ELC-Cu(II)-induced meiotic arrest**

(A) qRT-PCR analysis of Lipt1 mRNA levels in mouse oocytes to validate knockdown efficiency. (B)Representative images of oocyte maturation in control and lipt1-knockdown (lipt1-KD) groups cultured in medium containing 0.6 μM ELC-Cu(II). Scale bar = 100 μm. (C) Quantification of polar body extrusion (PBE) rates. Data are presented as mean ± SEM. Sample sizes: n = 100 (Control) and n = 103 (NC-KD). ***P < 0.001.
